# Abnormal Innate Immunity in Acute-on-Chronic Liver Failure: Immunotargets for Therapeutics

**DOI:** 10.3389/fimmu.2020.02013

**Published:** 2020-10-08

**Authors:** Arshi Khanam, Shyam Kottilil

**Affiliations:** Division of Clinical Care and Research, Institute of Human Virology, University of Maryland School of Medicine, Baltimore, MD, United States

**Keywords:** acute-on-chronic liver failure, innate immune cells, cytokines, liver damage, immune targets

## Abstract

Acute-on-chronic liver failure (ACLF) is a severe life-threatening condition with high risk of multiorgan failure, sepsis, and mortality. ACLF activates a multifaceted interplay of both innate and adaptive immune response in the host which governs the overall outcome. Innate immune cells recognize the conserved elements of microbial and viral origin, both to extort instant defense by transforming into diverse modules of effector responses and to generate long-lasting immunity but can also trigger a massive intrahepatic immune inflammatory response. Acute insult results in the activation of innate immune cells which provokes cytokine and chemokine cascade and subsequently initiates aggressive systemic inflammatory response syndrome, hepatic damage, and high mortality in ACLF. Dysregulated innate immune response not only plays a critical role in disease progression but also potentially correlates with clinical disease severity indices including Child-Turcotte-Pugh, a model for end-stage liver disease, and sequential organ failure assessment score. A better understanding of the pathophysiological basis of the disease and precise immune mechanisms associated with liver injury offers a novel approach for the development of new and efficient therapies to treat this severely ill entity. Immunotherapies could be helpful in targeting immune-mediated organ damage which may constrain progression toward liver failure and eventually reduce the requirement for liver transplantation. Here, in this review we discuss the defects of different innate immune cells in ACLF which updates the current knowledge of innate immune response and provide potential targets for new therapeutic interventions.

## Introduction

Acute-on-chronic liver failure (ACLF) is defined as acute decompensation of a patient with cirrhosis, associated with an intra- or extra-hepatic precipitating event resulting in one or more extrahepatic organ failures and high short term mortality (1). The Asian Pacific Association for the Study of the Liver (APASL) characterized ACLF as acute hepatic insult manifesting as jaundice and coagulopathy, complicated within 4 weeks by ascites and/or encephalopathy in a patient with previously diagnosed or undiagnosed chronic liver disease (CLD) (2). Later on the European Association for the Study of Liver's (EASL) Chronic Liver Failure (CLIF) Consortium Acute-on-Chronic Liver Failure in Cirrhosis (CANONIC) study elaborated the ACLF definition by including high 28-day mortality and established diagnostic criteria (1).

The presence of existing chronic liver disease distinguishes ACLF from acute liver failure (ALF) where no chronic liver disease prevails (3). Unlike ACLF, ALF is unpredicted and has a rapid onset that occurs most often in patients who do not have preexisting liver disease and can evolve over days or weeks to a lethal outcome or mortality (4). A variety of hepatic insults including viral and autoimmune hepatitis, drug induced liver injury, paracetamol cytotoxicity, hepatic ischemia, and herbal and dietary supplements and several other insults result in the rapid onset of clinical manifestations in ALF (3, 5). Despite having diverse causes, ALF is characterized by similar clinical features. The clinical presentation generally includes hepatic dysfunction, abnormal liver biochemical values, coagulopathy, and hepatic encephalopathy with multiorgan failure leading to death (3). A common feature which is shared by all etiologies of ALF is the hepatocellular loss of magnitude and at a rate which surpasses the liver regenerative capacity (6). The mechanism of liver injury contributing to ALF can be recapitulated into two categories: direct damage and immune-mediated liver injury (7). Various pathogens and toxic substances directly induce hepatic damage or trigger cell signaling cascade pathways and disturbs intracellular homeostasis. However, suitable intervention for these pathways could protect against ALF (8, 9). Immune-mediated liver injury particularly includes the innate and adaptive immune system and the contribution of cytokines. However, the innate immune system has a more intense contribution than the adaptive immune system as it gets activated more rapidly compared with adaptive immunity, mainly in the case of acute liver injury where the host has little time to trigger effective adaptive immune responses. A vigorous innate immune response induces hepatocyte death and liver injury which drives many other clinical features of ALF.

Though, the precipitating event in ACLF are mostly similar to ALF including drug-induced liver injury (10), viral (11), or autoimmune hepatitis (12), and alcohol-related liver injury. Other intra- and extra-hepatic factors also contribute to acute insult in ACLF including hypoxia injury and liver surgeries including transjugular intrahepatic portosystemic shunt placement and bacterial infections (13, 14). Unrecognized precipitating events also participate in acute insult leading to ACLF. However, both the condition including ACLF and ALF are reversible, if the acute insult is well-identified and specific intensive care support is given.

## Innate Immune Activation During CLD

Organ-specific and systemic inflammation are the essential events in the development and course of CLD (15), where innate and adaptive immune dysfunction play a pivotal role in the disease pathogeneses. Like acute insult, immune activation in CLD evolves from chronic liver injury of any etiology, most commonly viral infections, alcohol abuse, or hepatotoxic drugs that cause the activation of resident as well as infiltrating immune cells leading to disease progression toward fibrosis and cirrhosis, which disturbs the normal hepatic architecture and function, eventually leading to ACLF, in response to an acute insult.

During CLD, immune dysfunction mainly involves the components of the innate immune system which are distributed throughout the circulation as well as the tissue compartment and are critical to maintain homeostasis by preventing microbial invasion. Monocytes, macrophages, and neutrophils infiltration is a major contributing factor for CLD. These cells detect tissue damage and invading microorganisms and facilitate tissue healing and eradication of the infection. However, they also mediate intrahepatic and systemic inflammation by elevating inflammatory immune mediators through responding to damage-associated molecular patterns released from the injured cells (16), which further exacerbates an acute insult. Monocytes are critical for the commencement and perpetuation of tissue damage, providing the base for CLD. The frequency of non-classical monocytes has been shown to increase in early stages of CLD. These monocytes were preferentially accumulated in the liver and retained restricted phagocytic activity with elevated proinflammatory cytokines production (17–19). Intrahepatic neutrophil accumulation is a protuberant histological feature of CLD (20, 21); though, its contribution is not much studied. Abnormal neutrophil adherence capacity have been reported in patients with CLD (22), irrespective of the underlying etiology. Whereas, phagocytic activity and killing mechanisms remained normal (23). Similarly, hepatic macrophages perform diverse functions in homeostasis, progression and regression of CLD, and hold the central position as a potential target in combatting CLD. The progression of CLD from hepatitis to fibrosis and eventually cirrhosis is closely related to an enrichment of CD14+CD16+ monocyte-derived macrophages in the liver of patients with various disease etiologies (18). Freshly infiltrating hepatic macrophages boost chronic liver injury and fibrosis through transforming growth factor-β (TGF-β)-mediated hepatic stellate cell proliferation and transdifferentiation (24). Monocyte and macrophage activation are the part of inflammatory cascade during CLD. Activation of these cells in response to endotoxin leads to the upregulation of proinflammatory cytokines such as tumor necrosis factor-alfa (TNF-α) and IL-6 which induces inflammation (25). Spontaneous and lipopolysaccharide (LPS)-induced TNF-α production has been shown to be intensified even at the early stages of CLD (26, 27). The pivotal role of macrophages in inflammation and fibrosis during CLD has increased the interest for systemic markers of macrophage activation. Soluble CD163 (sCD163) has been identified as a specific macrophage activation marker associated with the severity of liver disease (28) and provides a potential target for therapeutics. Few studies have also reported the role of innate lymphoid cells (ILCs) in CLD progression (29, 30). ILCs are the most recently discovered group of innate immune cells that originated from common lymphoid progenitors and function through the secretion of signaling molecules, and the regulation of both innate and adaptive immune cells. ILCs are abundantly present in the liver with the dominant ILCs subset being cytotoxic natural killer (cNK) cells and ILC1s (31). Although, ILC2s and ILC3s are also present in the liver but they are infrequent. Liver injury during CLD has been significantly attributed to enhanced ILCs response, as reflected by markedly evaluated levels of IFN-γ, IL-12, and T-bet signaling (29). Dysregulation of ILCs can cause severe hepatic inflammation. Similarly, IL-13 produced by ILCs have a prominent role during CLD (32). A group of IL-33-dependent hepatic ILCs induced hepatic fibrosis via an IL-13-dependent mechanism which suggests the profibrogenic properties of ILCs (33). ILCs recruit eosinophils through IL-5 and amplify inflammation during autoimmune hepatitis. Taken together, these studies suggest a pivotal role for innate immune cells during CLD that hold great promise for immunotherapeutics.

## Innate Immune Activation During ACLF

**Figure 1:**
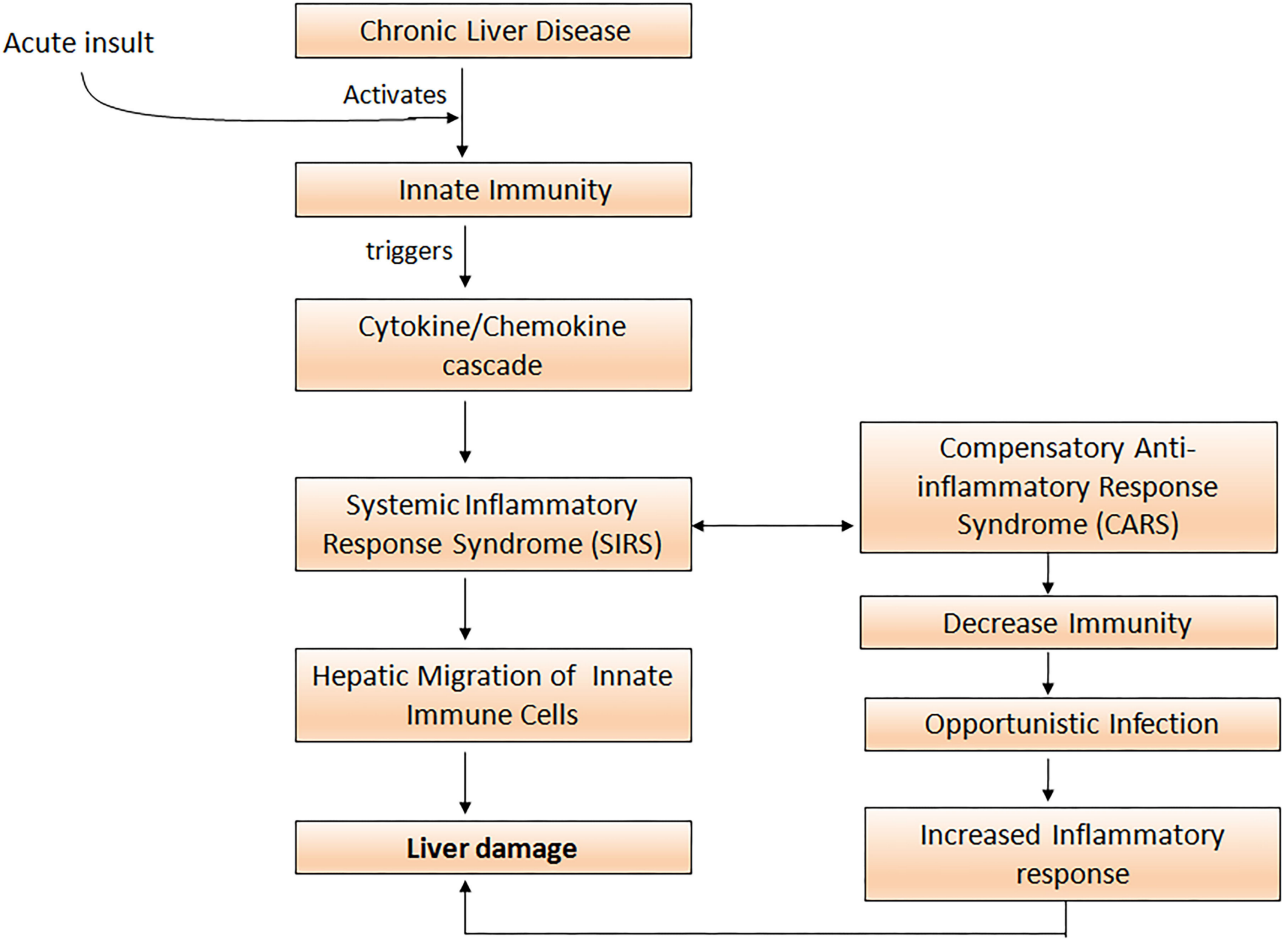
Acute insult in ACLF patients activates innate immune cells which elicit cytokine and chemokine cascade resulting in the initiation of the systemic inflammatory response (SIRS). To overcome SIRS, compensatory anti-inflammatory responses increase susceptibility to opportunistic infections and further aggravate the pro-inflammatory response leading to severe liver damage.

Uncontrolled activation of both the innate as well as the adaptive immune system is a prerequisite for the development of systemic inflammation in ACLF (34). Cytokines and chemokines are critically involved in the process of leukocyte recruitment and activation in the liver, and therefore may perpetuate liver damage by extending inflammatory pathways and compromising other organs (35). Acute insult in ACLF leads to the activation of innate immune cells which trigger inflammatory cytokine cascade and initiates aggressive systemic inflammatory response syndrome (SIRS). Subsequently, to overcome SIRS the development of compensatory anti-inflammatory response (CARS) takes place. With the development of CARS, patients are prone to acquire infection that would further exacerbate pro-inflammatory responses, resulting in massive hepatic damage and high mortality (Figure 1).

In ACLF, functional impairment of almost all types of immune cells occurs. Few studies have endeavored to diagnose early events correctly to identify the role of the innate immune response in ACLF patients (36). The innate immune response typically depends on the cells that do not need any additional training to perform their function and can cause tremendous systemic as well as organ specific inflammatory responses rapidly. These cells include neutrophil granulocytes, monocytes, macrophages, dendritic cells (DCs), myeloid derived suppressor cells (MDSCs), and natural killer (NK) cells. An innate immune response arises quickly after acquiring any kind of infection through a disease-causing pathogen or any other harmful foreign substances. Neutrophils, monocytes, and macrophages ingest the invading microorganisms, foreign molecules, and dying cells through their phagocytic activity and help in the resolution of infection. Monocytes and dendritic cells activate adaptive immunity through antigen presentation. NK cells control several types of microbial infection by limiting their spread but also encourage tissue damage. The current review will provide insights on the defects of innate immune cells and highlight numerous propitious immune targets for the treatment and management of ACLF patients.

## Defects In Innate Immune Cells Of ACLF

**Figure 2:**
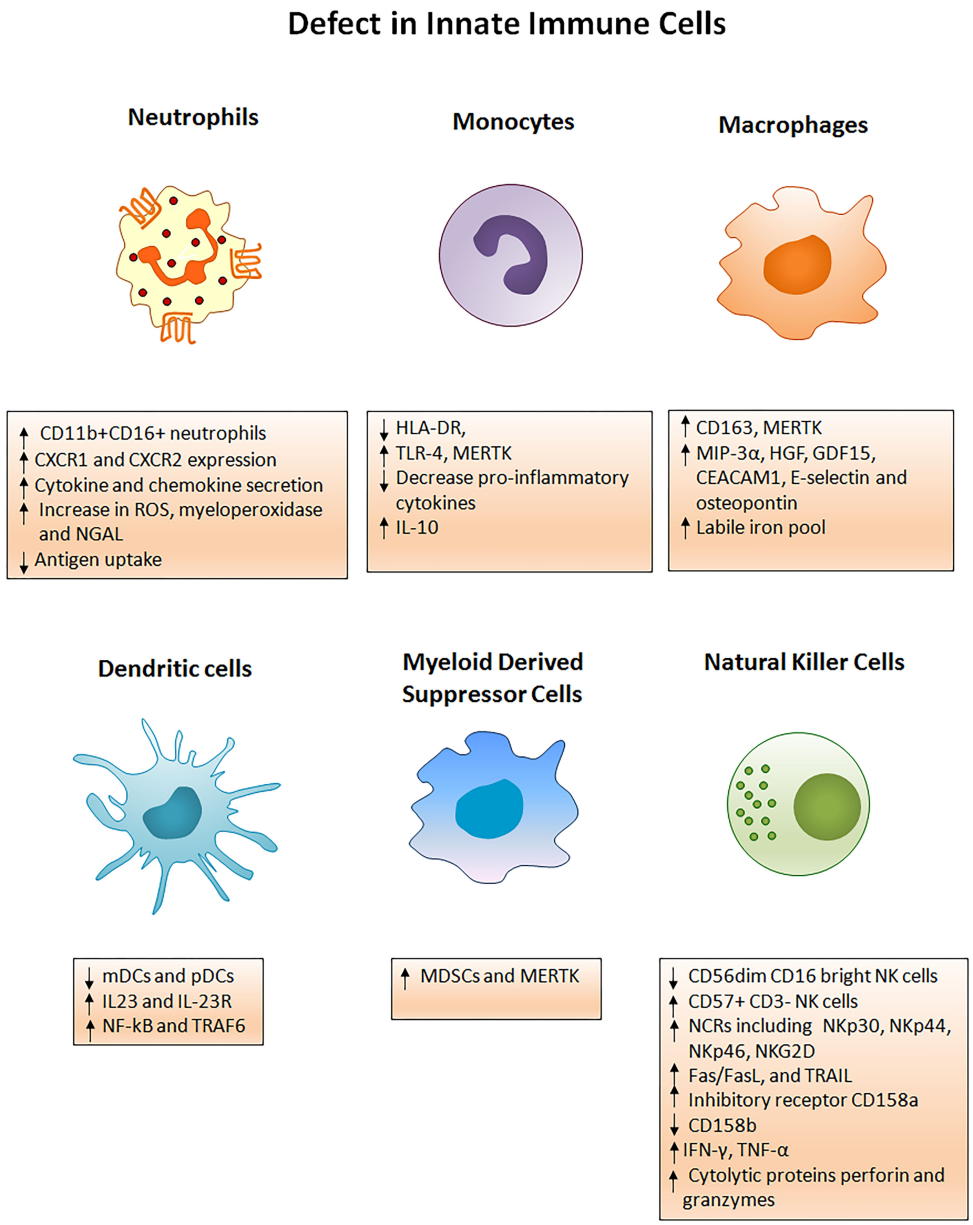
Innate immune mechanisms of liver injury. Several phenotypic and functional defects in cellular components of the innate immune system occur in ACLF. Any acute insult (virus, alcohol, drug, autoimmune hepatitis, or cryptogenic) causes the hyper activation of innate immune cells through several receptors present on the cell surface. Activation of the cells results in cell specific consequences such as secretion of pro/anti-inflammatory cytokines from monocytes, neutrophils, macrophages, DCs, MDSCs, and NK cells that promote liver immunopathology. Neutrophils start producing huge amounts of reactive oxygen species whereas NK cells secrete abundant cytotoxic granules which cause oxidative stress and hepatoxicity. Decreased HLA-DR expression on monocytes makes them inefficient in antigen presentation. ROS, Reactive oxygen species; NGAL, Neutrophil gelatinase associated lipocalin; HLA-DR, Human leukocyte antigen; TLR4, Toll-like receptor 4; MERTK, MER tyrosine kinase; IL, Interleukin; MIP-3α, Macrophage inflammatory protein 3α; HGF, Hepatocyte growth factor; GDF15, Growth differentiation factor 15; CEACAM, Carcinoembryonic antigen-related cell adhesion molecule; mDCs, Myeloid dendritic cells; pDCs, Plasmacytoid dendritic cells; MDSCs, Myeloid derived suppressor cells; NF-kB, Nuclear factor kappa B; TRAF6, TNF receptor associated factor; NK cells, Natural killer cells; NCRs, Natural cytotoxic receptor; TRAIL, TNF related apoptosis inducing ligand; IFN-γ, Interferon gamma; TNF-α, Tumor necrosis factor- α.

Immune defects in ACLF are multifactorial and involve abnormalities in cellular and soluble components of the immune system. Cellular components involve functionally reprogrammed innate and adaptive immune cells; while soluble components include cytokines, chemokines, and complement molecules. Here, we are discussing defects in the innate immune cells. Figure 2 briefly illustrates different phenotypic and functional abnormalities in innate immune cells of ACLF patients that have been elaborated below.

## Neutrophils

Neutrophils, also known as polymorphonuclear granulocytes, are the major players of the innate immune system. They are typically the first cells to be recruited to the site of infection where they provide defense against invading pathogens by eliminating them through multiple intracellular and extracellular mechanisms (37). Apart from removing the pathogen, neutrophils release an array of inflammatory mediators such as serine proteases (38, 39), elastases, cathepsin G (39), nicotinamide adenine dinucleotide phosphate (NADPH) oxidases (40), myeloperoxidases (MPO) (41), and matrix metalloproteases (37) essential to control localized infection but can also facilitate tissue damage (42). Data obtained from both experimental as well as clinical studies showed that, following infection, neutrophil migration to the inflammatory site is indispensable for the clearance of infection (43, 44). Indeed, decreased circulating neutrophil numbers leads to severe immunodeficiency in humans predisposed to bacterial and fungal infections (45). Clinical use of granulocyte-colony stimulating factor therapy enhanced neutrophil function in ACLF patients (APASL ACLF) and prevented the development of sepsis and decreased disease severity indices including Child-Turcotte-Pugh (CTP), the model of end stage liver disease (MELD), and the sequential organ failure assessment (SOFA) score (46). Impaired neutrophil function has also been attributed as an important biomarker in predicting the outcome of alcoholic hepatitis (47). Other data revealed that impaired neutrophil function contributes to liver injury and is associated with clinical disease severity indices in ACLF (48).

**Figure 3:**
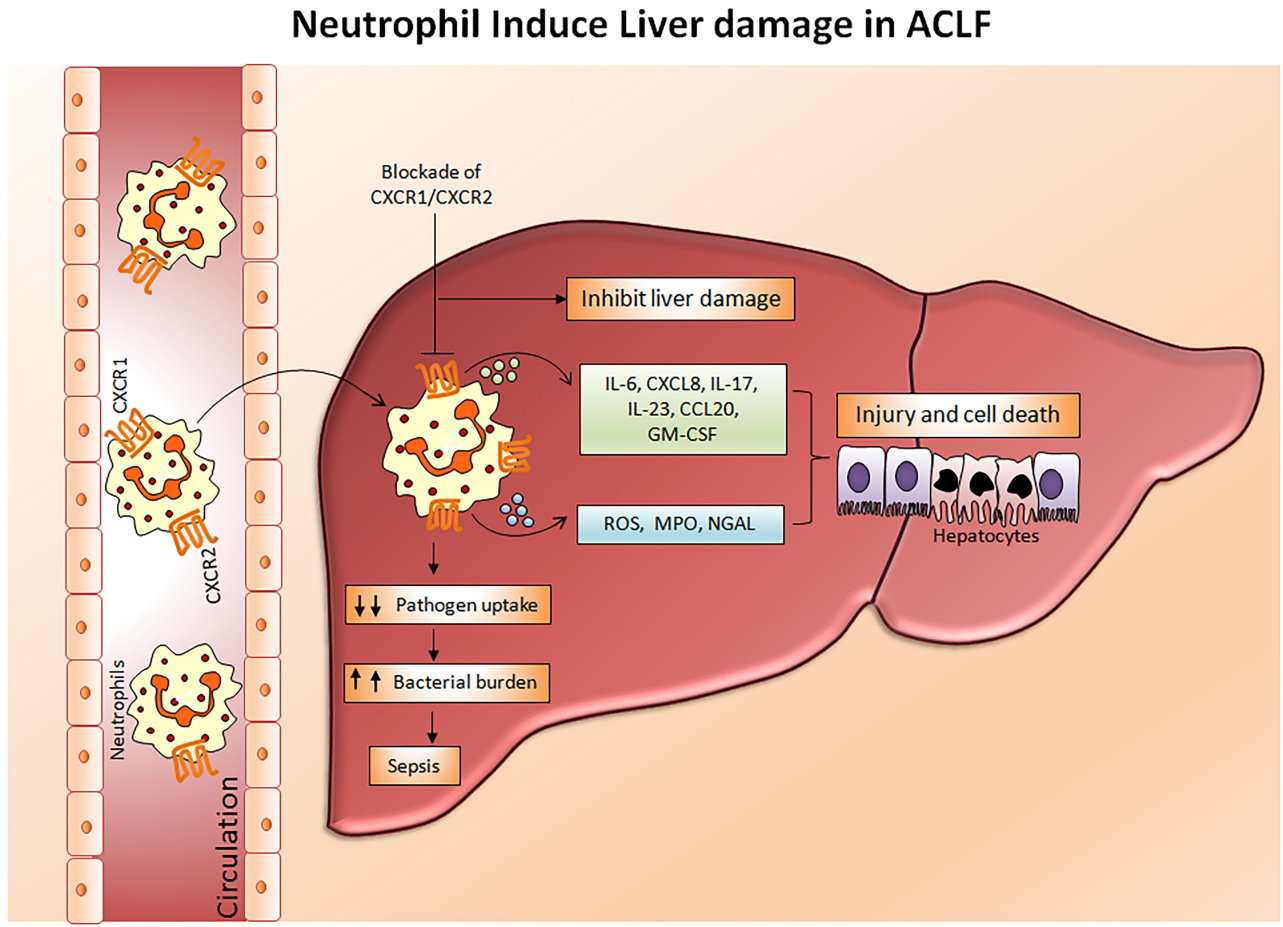
Mechanism of neutrophil mediated hepatocyte death. In ACLF, a hyper activation of neutrophils occurs which results in a dramatic increase in pro-inflammatory cytokines, reactive oxygen species, and myeloperoxidases. Subsequently, with the help of chemokine receptors CXCR1/CXCR2, circulating neutrophils migrate toward the liver where they produce abundant inflammatory mediators leading to liver injury and cell death which further aggravates hepatocellular damage. Impaired neutrophil phagocytic activity may direct the development of sepsis by increasing bacterial burden. Blockades of CXCR1/CXCR2 have immense potential to inhibit neutrophil mediated hepatocyte death. CXCR, Chemokine receptor; IL, Interleukin; CCL-20, Chemokine ligand 20; GM-CSF, Granulocyte macrophage colony stimulating factor; ROS, Reactive oxygen species; MPO, Myeloperoxidase; NGAL, Neutrophil gelatinase associated lipocalin.

Chemokine receptors CXCR1 and CXCR2 have been shown to mediate neutrophil response to CXC chemokines primarily CXCL8, produced by several hepatic cells including hepatocytes, endothelial cells, stellate cells, and immune cells comprising macrophages, (49) Kupffer cells, and Th17 cells, expediting neutrophil migration to the liver. CXCL8, also known as the neutrophil chemotactic factor, induces a series of physiological responses required to intercede neutrophil chemotaxis, and transendothelial migration and activation. Primarily CXCL8 encourages neutrophil migration to the liver, where they promote phagocytosis to clear the infection (50). CXCR1 and CXCR2 have different binding affinities. CXCR1 have a high binding affinity to CXCL8 whereas CXCR2 can bind to CXCL1, CXCL2, CXCL8, and CXCL9. Lower CXCR1/2 expression on CD66b+ neutrophils in hepatitis B virus-related ACLF (HBV-ACLF) patients (APASL ACLF) has been served as a prognostic marker for disease severity and outcome (51). Whereas, increased intrahepatic and circulating CXCL8 is found to be responsible for liver inflammation and reduced CXCR1/2 expression, respectively. High CXCR1/2 expressing CD11b+ and CD16+ neutrophils induce hepatocyte death in ACLF patients through contact-dependent as well as contact-independent mechanisms (52). CXCR1/2 expands early apoptosis and necrosis of hepatocytes leading to cell death and blockade of CXCR1/2 with antagonist SCH527123 significantly diminished cell death (53). Also, CXCR1/2 regulates the production of inflammatory cytokines/chemokines including IL-6, IL-17A, IL-23, CXCL8, CCL20, and GM-CSF, MPO and reactive oxygen species (ROS) in ACLF patients (APASL ACLF) which causes oxidative stress and subsequently induces cell death (53, 54). Moreover, the chemokine ligand CXCL8 produced by the neutrophils also mediates neutrophil activation and infiltration through CXCR1 and CXCR2 and aggravates liver injury in ACLF patients (55). Therefore, targeting CXCR1/2 not only offers protection against direct contact-dependent hepatocyte death but also decreases the production of inflammatory mediators and ROS, accountable for cell death (Figure 3). Investigations also emphasized that alcohol-induced liver injury is neutrophil mediated and CXCR1/2 signaling plays a critical role in a murine model of alcoholic steatohepatitis (ASH). The blockade of CXCR1/2 receptors with pepducins increases survival and regresses hepatic inflammation and steatosis (56). Interestingly, blocking CXCR1/2 with pepducins does not impede neutrophil chemotaxis, induced by other neutrophil chemoattractants, thus pepducins may be considered to have an immunomodulatory rather than an immunosuppressive role. Furthermore, CXCR1/2 blockade suppresses SIRS in a murine model of sepsis without yielding higher bacterial load (57). Systemic inflammation is known as a hallmark of ACLF (58); hence the inhibition of CXCR1/2 could prevent systemic inflammation and further development of sepsis. In acetaminophen-induced liver injury, CXCR2 regulates neutrophil infiltration that aggravates systemic inflammation leading to severe organ damage, CXCR2 inhibition potentially restricts hepatotoxicity (59). On the contrary, other studies established that neutrophils do not contribute to acetaminophen-induced liver injury (60, 61).

Liver cell necrosis promotes the secretion of cytokines which later initiates the innate immune response and leads to an abundant release of granulocytes from bone marrow to peripheral blood consequently decreasing the lymphocyte number. The neutrophil to lymphocyte ratio (NLR) is an easily measured marker to evaluate systemic inflammation (62). Recently, NLR has been utilized as an independent predictor of poor prognosis and short term mortality in HBV-ACLF (APASL ACLF) (63, 64). Patients who had NLR ≥ 3 had lower mortality; however, those with NLR > 6 were at greater risk of high mortality. Kaplan-Meier analyses also demonstrated that each increase in NLR is associated with higher 30-day mortality. High NLR has also been utilized as a predictor of prognosis in ACLF patients (APASL ACLF) after liver transplantation (65). ACLF patients admitted to the intensive care unit exhibiting higher NLR predict death in comparison to cirrhotic patients; in fact, ACLF non-survivors displayed a higher NLR ratio. NLR was not only found to be a predictor of mortality, but also suggested the sequential organ failure assessment score and mechanical ventilation requirement in ACLF patients (EASL-CLIF ACLF) (66).

Impaired neutrophil function enables nosocomial infections (67). Patients with severely impaired neutrophil function are at maximum risk of developing these infections (68). Therefore, it would not be an exaggeration to say that impaired neutrophil functions are accountable for secondary infections and subsequent sepsis development in ACLF patients. Neutrophils release abundant neutrophil gelatinase-associated lipocalin (NGAL) also known as lipocalin-2 that plays a vital role in innate immunity as well as various pathological conditions (69). NGAL is synthesized as a component of late granules of neutrophils, where it colocalizes with myeloperoxidase and can regulate organ damage, inflammation, metabolism, and tumorigenesis. In the case of the liver, NGAL appears to have both a protective as well as a pathogenic role (70, 71). NGAL gets upregulated in an experimental model of liver injury due to damage caused by toxins and inflammatory cytokines (72). An increased level of urine NGAL has been seen as an independent predictive factor of 28-day mortality and improved the accuracy of MELD in predicting prognosis. Moreover, increased intrahepatic NGAL gene expression correlated with serum bilirubin, the INR and MELD score, and was associated with systemic inflammation and liver failure in ACLF patients (EASL-CLIF ACLF) (73).

## Monocytes

**Figure 4:**
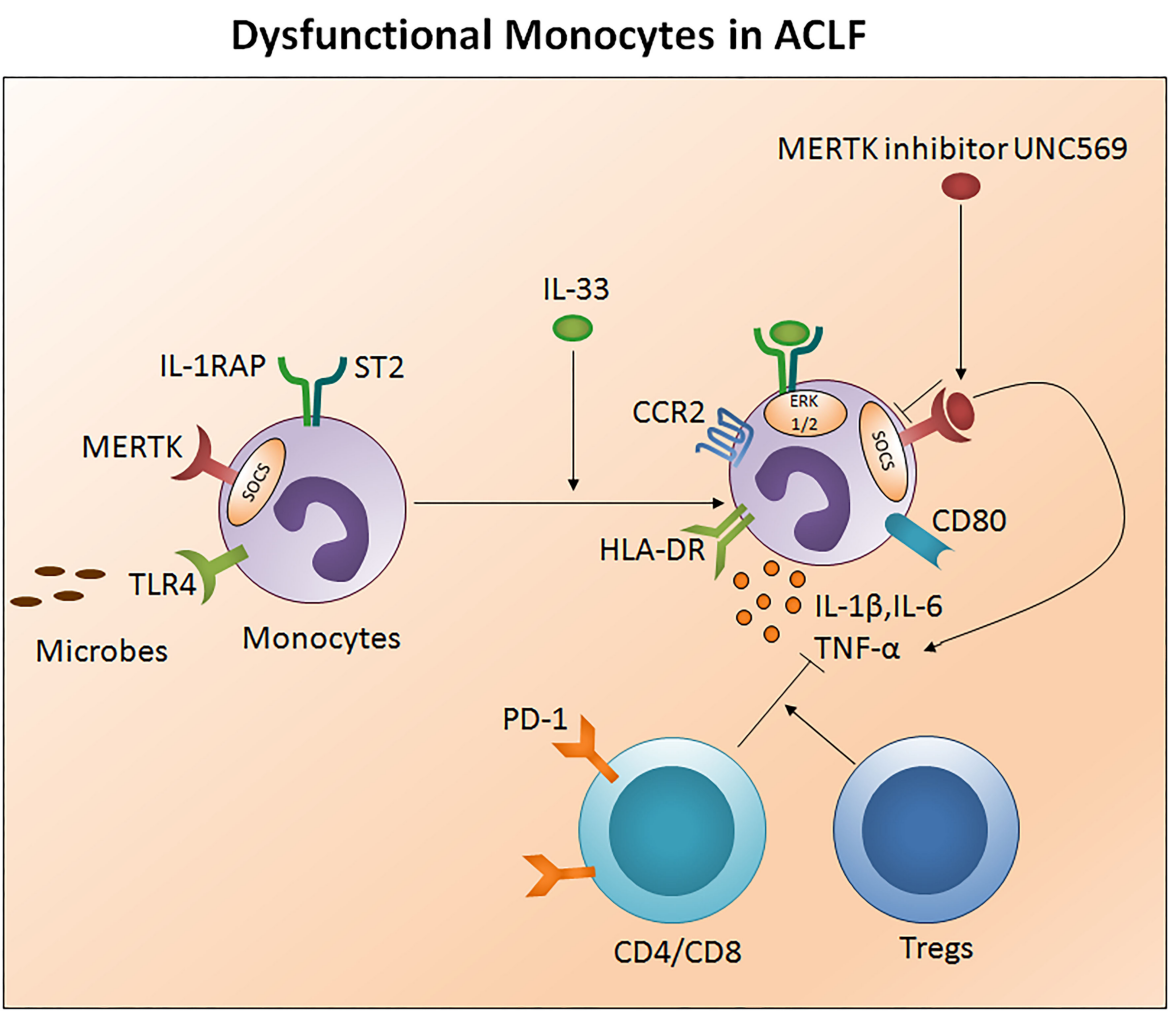
Impaired monocytes function in ACLF. Monocytes present antigens through human leukocyte antigen class II molecules such as HLA-DR. Ongoing injury in ACLF decreases monocyte HLA-DR expression, escalates MERTK expression, and subsequently impairs monocyte function to control the infection. MERTK inhibits the monocyte inflammatory response through avoiding TLR activation and inflammatory cytokine production. High PD1 expressing CD4/CD8 T cells and Tregs constrain monocyte inflammatory cytokine production. IL-33 re-establishes monocyte HLA-DR expression along with CCR2 and the co-stimulatory molecule CD80 and restores inflammatory cytokine production through the activation of the ERK1/2 pathway. MERTK blockades inhibit SOCS signaling and improve the production of inflammatory cytokines. HLA-DR, Human leukocyte antigen- antigen D related; MERTK, MER tyrosine kinase; TLR, Toll-like receptor; PD-1, Programmed death-1; CCR2, Chemokine receptor 2; ERK, Extracellular signal-regulated kinases; SCOS, Suppressor of cytokine signaling.

Monocytes are well-known to originate in the bone marrow from common hematological myeloid precursors and then get released into peripheral blood where they circulate for several days before entering tissues. Monocytes have the capacity to differentiate into macrophages and myeloid lineage dendritic cells. Monocytes and neutrophils appear early at the site of infection to encourage innate immune responses. They present antigens through human leukocyte antigen (HLA) class II molecules and produce large quantities of pro-inflammatory cytokines. Low HLA-DR expression was observed in early stage ACLF patients which was even lower in the late stage. Importantly both early and late stage ACLF non-survivors had lower HLA-DR expression as compare to those who survived. Decreased HLA-DR expression has been shown to be associated with high mortality rates in ACLF (74). In fact, lower HLA-DR expression showed positive correlation with prothrombin time which is one of the most important indexes for the measurement of hepatic injury (74). A reduction in HLA-DR expression may signify the physiological downregulation of monocyte function to control overstimulation in response to an acute hyper inflammatory response, and later adapted immunodeficiency that inclines recurrent infection resulting in an increased risk of death. In addition, monocytes express high TLR-4 in early stage ACLF and produce higher amounts of pro-inflammatory cytokines including IL-1β, TNF-α, and IL-12p70 and less anti-inflammatory IL-10, which increased during the later stage. A rise in both pro- as well as anti-inflammatory cytokines suggests a complex interplay between pro- and anti-inflammatory responses in ACLF (74). IL-33 restores monocytes HLA-DR expression along with chemokine receptor 2 and CD80 (75) and augmented monocyte inflammatory cytokines comprising IL-6, IL-1β, and TNF-α through activation of the ERK1/2 pathway without hampering monocyte phagocytic activity (Figure 4).

Monocyte dysfunction is linked with high MER receptor tyrosine kinase (MERTK) expression in the circulation, liver, and lymph nodes of ACLF patients (CANONIC ACLF) (76). Notably, augmented MERTK expression correlates with hepatic as well as extrahepatic diseases and systemic inflammatory responses. MERTK is a transmembrane protein of the tyro, AXL, and MER-receptor family expressed on monocytes and other cells including macrophages, dendritic cells, and epithelial cells which negatively regulate the innate immune response and help to resolve inflammation through the inhibition of pro-inflammatory responses. In the case of monocytes, MERTK regulates the inflammatory response by preventing TLR activation and pro-inflammatory cytokine production through the activation of suppressors of cytokine signaling. Interestingly, a high MERTK expression reduces the monocyte response to lipopolysaccharide. However, the addition of UNC569, a MERTK inhibitor, restores the production of inflammatory cytokines in response to LPS (76). Therefore, MERTK inhibitors could provide a powerful tool to restore monocytes function to circumvent repetitive microbial infections in ACLF. Also, CD14+HLADR- monocytic myeloid-derived suppressor cells (M-MDSCs) decreased T cell function and the antimicrobial innate immune response in ACLF (EASL-CLIF ACLF) which was associated with secondary infection, disease severity, and prognosis (27). Recently, it has been established that ACLF patients have a discrete transcriptional profile of monocytes which are polarized toward an immunotolerant state and altered metabolism. Transcriptional profiling of isolated CD14^+^CD16^−^ monocytes disclosed an upregulation of immunosuppressive parameters and compromised antibacterial and antigen presentation machinery. Moreover, the glutamine synthetase/glutaminase ratio of ACLF (EASL-CLIF ACLF) in patient-derived monocytes showed a positive association with disease severity scores including MELD. Remarkably, metabolic rewiring of the cells using a pharmacological inhibitor of glutamine synthetase, partially restored the inflammatory and phagocytic capacity of *in vitro*-generated as well as ACLF patients-derived monocytes and highlights its biological relevance (77).

A decreased ratio of CD3+ cells and monocytes (T/M) has been reported in the early to intermediate stage of ACLF which reached its lowest at the last stage; however an increased T/M ratio was seen in patients with good prognosis compared to patients with poor outcomes. An increase in programmed death 1 (PD-1) receptor expression in both CD4+ and CD8+ cells resulted in the loss of T cells in these patients. Both CD4 and CD8 T cells along with regulatory T cells tempered the monocyte innate immune function by inhibiting monocyte TNF-α secretion resulting in dysfunctional monocytes (78).

## Macrophages/Kupffer Cells (KCs)

Macrophages are essential components of the innate immune system and are present in most tissue types. Earlier macrophages were mainly known to be involved in host defense and elimination of apoptotic cells/bodies, however emerging evidence characterized macrophages as multifunctional cell types that are also implicated in tissue development, homeostasis, repair (79), and regeneration (80). Depending on the microenvironmental signals including activated lymphocytes, microbial products, and damaged cells, macrophages differentiate into variable types with a diverse functionality. Macrophages polarize into classically activated (M1) and alternatively activated (M2) macrophages (81). Both macrophage populations are functionally different: the M1 phenotype typical function includes high antigen presentation, production of inflammatory cytokines TNF-α, IL-1, IL-6, IL-12, Type I IFN, CXCL1-3, CXCL-5, and CXCL8-10, ROS, and nitric oxide (49). Conversely, M2 macrophages exhibit a resting phenotype, express mannose receptors, scavenger receptor A and B-1, CD163, CCR2, and are involve in tissue healing (82). M2 macrophages typically produce anti-inflammatory cytokines IL-4, IL-10, and IL-13 (79) along with ornithine and polyamines which play an essential role in diverse cellular responses. It has been reported that at an early stage of ACLF (EASL-CLIF ACLF) macrophages release TNF-α and IL-6, while later they release IL-10 (36). TNF-α is especially critical in the pathogenesis of liver dysfunction and correlates with the severity of liver injury and the development of SIRS.

Recently, it has been demonstrated that TNF-α promotes the apoptosis of resident liver macrophages, also known as Kupffer cells, by increasing the expression level of large non-coding RNA hypoxia-inducible factor 1 alpha-antisense RNA (HIF1A-AS1). Silencing the expression of HIF1A-AS1 reduced TNF-α induced KCs apoptosis (83) and provide a molecular basis for the clinical treatment of ACLF. Kupffer cells constitute more than 80% of the body macrophage population and play a crucial role in liver inflammation and fibrosis (84). Therefore, liver macrophages could be involved in the development and progression of clinical complications in cirrhosis including ACLF. Various stimuli could activate macrophages, though in ACLF, pathogen associated molecular patterns (PAMPs) appears to be a major contributor (85). Disruption of the gut-blood barrier promotes the translocation of gut bacteria and PAMPs to the liver and activates KCs via the induction of toll-like receptors, resulting in the secretion of a number of cytokines which leads to hepatic as well as systemic inflammation (35). Activated macrophages shed the hemoglobin-haptoglobin scavenger receptor CD163 into the circulation as sCD163. ACLF patients (EASL-CLIF ACLF) showed a stepwise increase in sCD163 with rising grades of ACLF and confirmed an independent association with short as well as long term mortality (86). sCD163 had a strong correlation with disease severity including, fibrosis, cirrhosis, CTP score, portal hypertension, and MELD scores and had equal or even higher predictive accuracy than MELD, CLIF-C, and CLIF-C AD scores. In addition to sCD163, a soluble mannose receptor (sMR) was found highly increased in ACLF and introduced as a novel biomarker of macrophage activation (86). A mannose receptor is a C type lectin primarily presented on the surface of macrophages and dendritic cells; however other cells like hepatic cells, human dermal fibroblasts, lymphatic endothelia, and kidney cells also express the mannose receptor. Since sCD163 solely belongs to the monocytes/macrophage and similar changes were observed for sMR, the study suggests macrophages as a primary source of both sCD163 and sMR. The data further claimed that inflammatory activation of liver macrophages led to the development and progression of ACLF. An increase in M2 type macrophage markers including CD163, CD206, and TGM2 was seen in the liver of ACLF patients. Opposite to the previous study, these data demonstrated a significant association of increase in the CD163 and M2/M1 macrophage ratio with the magnitude of hepatic progenitor cell differentiation to hepatocytes and suggests that M2 macrophages encourage hepatocyte differentiation and hepatocyte progenitor cell-mediated liver regeneration in ACLF patients (APASL ACLF) (87). Macrophages also play a key role in iron homeostasis. M2 macrophages contain larger intracellular labile iron pools than M1 macrophages. Labile iron pool (LIP) is an intracellular pool of soluble, free, and redox-active iron which participates in pro-oxidant damage and is considered a critical pathogenic factor in various kinds of injury including the oxidative stress of hepatocytes. ACLF patients (APASL ACLF) have high LIP content in CD11b+ CD163+ macrophages (alternatively activated M1 macrophages) and CD163+ HLA-DR+ macrophages (mature phenotypes), which was even higher in patients with multiorgan failure. ACLF non-survivors had the highest LIP content which correlated with the MELD and SOFA scores (88). Similar to the monocytes, macrophages also exhibit high MERTK expression in ACLF patients (76). MERTK expressing peritoneal macrophages were higher than their circulating counterparts and were associated with the severity of intra- and extra-hepatic disease and systemic inflammatory response. Peritoneal MERTK+ macrophages exhibited an elevated expression of pro-resolution/anti-inflammatory tissue and lymph node homing receptors HLA-DR^high^CD163^high^CX3CR1^high^CCR7^high^ which contains remarkably similar phenotypic and functional similarities to their circulatory counterpart. Liver explants tissue also confirmed the major expansion of MERTK^high^ and CD163^high^ hepatic macrophages in ACLF which negatively regulated the immune response.

The pathological process in ACLF results in specific changes in the circulating protein level which could predict the outcome. Six proteins including macrophage inflammatory protein 3α (MIP-3α), hepatocyte growth factor, growth differentiation factor 15, carcinoembryonic antigen-related cell adhesion molecule 1, E-selectin, and osteopontin were found highly augmented in HBV-ACLF patients (89). Macrophages remains the major source of MIP-3α in the liver which helps in the recruitment of neutrophils, dendritic cells, and effector/memory T and B cells by utilizing the chemokine receptor 6 (CCR6). Crosstalk between MIP-3α and CCR6 might be crucial in the amplification of localized immune response in inflamed liver. Importantly, the MIP-3α level was greatly enhanced in the ACLF deceased group and correlated with 3-month mortality as compared to the survival group. A receiver operating curve also demonstrated MIP-3α as an independent predictor of mortality. However, MELD had the highest ROC-AUC, but when MELD and MIP-3α were combined, the prediction of mortality was improved, therefore, in place of using each indicator individually as a predicator of mortality, a combination of MELD and MIP-3α might be a better predictor of mortality in ACLF. Moreover, high serum MIP-3α showed no evidence of any harmful effect on the liver (90).

Currently, high HBV DNA has been shown to activate macrophages (91) that escalate the immune response against HBV through the production of inflammatory cytokines IL-6, IL-8, and TNF-α and limit infection by viral clearance. Previous studies performed on animal models including chimpanzees (92) and wood chucks (93) showed similar observations. The observation that high HBV DNA is required for the activation of macrophages are outstanding in the way that HBV infection proceeds without activating the immune system until its replication peaks in the infected hosts. Importantly, macrophages provokes HBV specific adaptive immunity in mouse models (94) and the depletion of macrophages decreases the recruitment of inflammatory cells and ameliorates liver injury (95). Macrophage response to high viral HBV DNA leads to rapid induction of TNF-α and IL-6 but the high-viremia required for macrophage activation is in line with the delayed pro-inflammatory cytokine production as well as the late onset of immune control. Studies performed on both animal models as well as humans revealed that macrophage activation happened after HBV infection, therefore we may consider similar working mechanisms of macrophages in HBV-ACLF patients.

## DCs

DCs are professional and versatile antigen-presenting cells that operate at the interface of the innate and adaptive immune system. To perform their functions, whether endorsing immunity to pathogens or in maintaining tissue tolerance, DCs require migration to the target site. Based on phenotypic and functional characterizations, two distinct circulating DCs subsets, myeloid, and plasmacytoid (mDCs and pDCs) have been identified in humans. DCs have been studied in HBV (96), HCV infections (97), and alcoholic liver diseases (98), however, few studies illustrated their role in ACLF.

Low frequencies of both mDCs as well as pDCs have been observed in ACLF patients (APASL ACLF) which was even lower in non-survivors. Lower DC numbers had a strong association with the CTP and SOFA scores and could be causative factors for high mortality. One-month therapy with a granulocyte colony-stimulating factor (G-CSF) enhanced the frequency of both type of DCs (99). Another study also revealed lower frequency as well as functionality of DCs in HBV-ACLF patients. Moreover, dead/transplanted ACLF patients had less mDCs functionality which was associated with high mortality. Notably, treatment with methylprednisolone resulted in continuous increase in mDCs accompanied by a marked reduction in total bilirubin and 28-day mortality (100). Baseline higher mDCs and subsequent recovery at the end of methylprednisolone therapy may represent a potential prognostic marker for a promising response to corticosteroid therapy in ACLF patients. In ACLF, monocytes derived dendritic cells (MoDCs) produce high amount of IL-23 and express its cognate receptor IL-23R. Higher IL-23 production in non-survivors are associated with MELD, INR, prothrombin time, and total bilirubin level and signify potential contribution in disease progression and severity (101). MoDCs showed upraised NF-κB and TNF receptor associated factor-6 (TRAF6) expression which was related to the elevated level of IL-23.

## MDSCs

MDSCs, who constitute the heterogeneous population of immature immune cells, belong to the myeloid lineage and have a great ability to suppress T cell responses. Under normal conditions, MDSCs regulate immune response and tissue repair; however, the population quickly expands during infection and inflammation. Numerous therapeutic strategies are now being evolved to target MDSCs to constrain immune responses in the setting of transplant rejection (102). Increased population of CD14^+^CD15^−^CD11b^+^HLA-DR^−^ M-MDSCs in ACLF patients occurred because of continuous immune stimulation from microbial and non-microbial inflammatory signals. These MDSCs exhibit immunosuppressive properties by declining T cell proliferation and less TNF-α and IL-6 production in response to TLR stimulation (27). Moreover, HBV surface antigens elicit the differentiation of monocytes toward M-MDSCs in an autocrine manner by the activation of the kinase ERK and transcription factor signal transducer and activator of transcription-3 (STAT-3) (103). Interestingly, MERTK also regulates the activation of myeloid cells in ACLF (EASL-CLIF ACLF) (104). The expansion of CD14^+^CD15^−^HLA-DR^−^ M-MDSCs in ACLF patients is also triggered by the activation of systemic inflammatory response syndrome and circulating pathogen associated molecular patterns (27). In addition to suppressing T cell activation, these M-MDSCs decrease the pathogen uptake and TLR elicited proinflammatory responses to microbial challenges. During disease progression, MDSCs persistence confers poor outcomes and are associated with an increased incidence of infection. Interestingly, TLR-3 agonism reversed the M-MDSCs expansion and innate immune function and merits additional evaluation as a potential therapeutic agent. In the advanced stage of ACLF, the expansion of circulating MDSCs has been observed; non-survivors retained the highest amount whereas the survival group displayed a gradual decline. MDSCs also showed a close association with ALT, TBil, INR level, and the MELD score (11).

## NK Cells

NK cells are cytotoxic effector lymphocytes of the innate immune system which control several types of microbial infections by limiting their spread, although hyper activation subsequently induces tissue damage. NK cells represent around 15% of the circulating blood lymphocytes and the proportion increases up to 30% in the liver. Generally, immune cells sense major histocompatibility complexes (MHC) presented on the surface of infected cells, initiating cytokine release and thus causing cell lysis or apoptosis. However, NK cells have the unique ability to identify stressed cells even in the absence of MHC which allows for a much faster immune reaction. This exceptional function of NK cells is quite important for those detrimental cells which miss the detection of MHC I markers and are destroyed by other immune cells.

**Figure 5:**
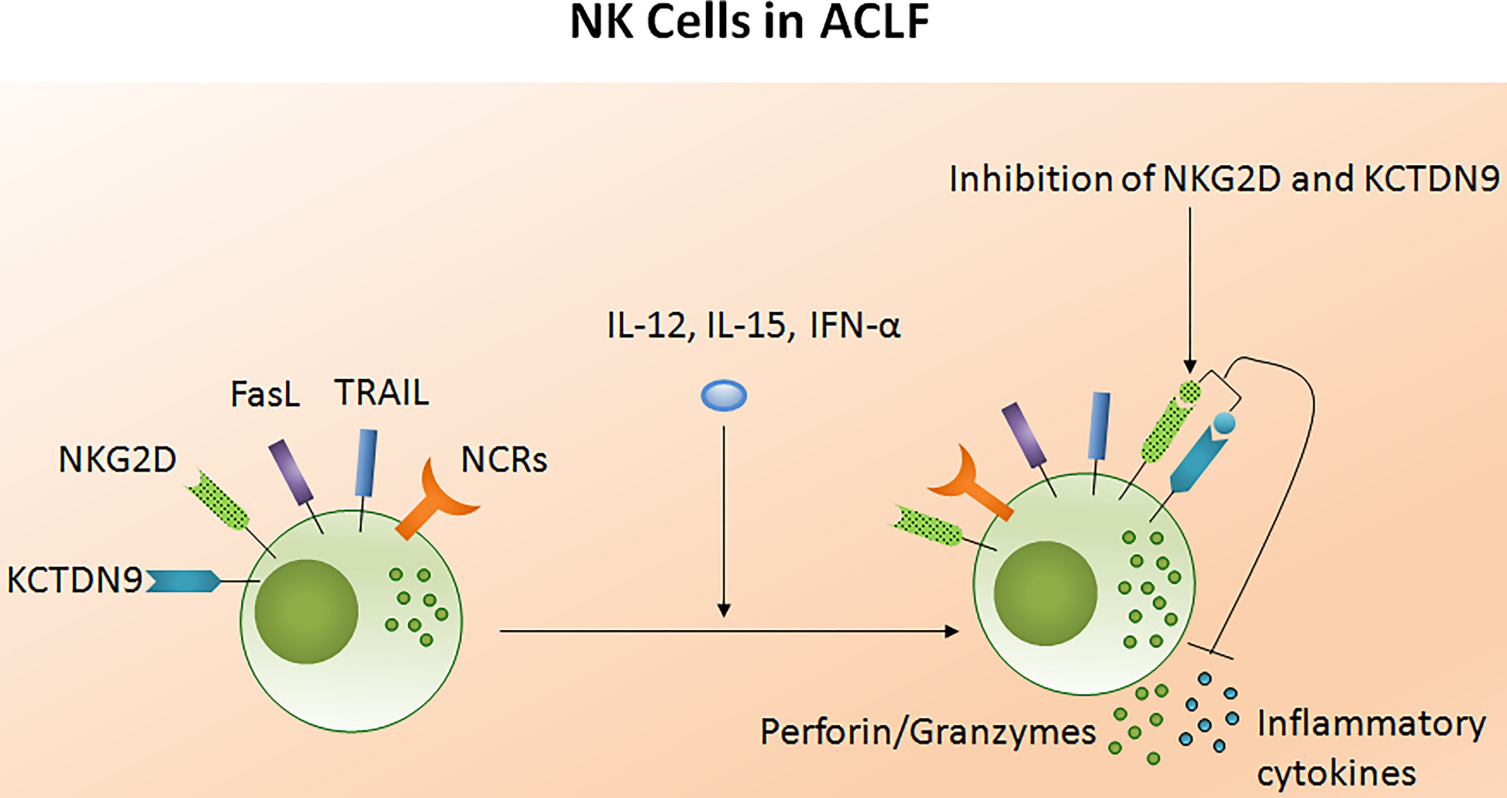
Liver injury causes NK cell dysfunction in ACLF. The functionality of NK cells is controlled by activating and inhibitory receptors. The expression of NCRs and inhibitory receptors are increased during ACLF; however cytotoxic activity and cytokine production decreases. Increased FASL, TRAIL, and NKG2D induced hepatocyte death through apoptosis and necrosis. NKG2D encourage liver inflammation whereas KCTD9 increases NK cell mediated cytotoxicity. Treatment of NK cells with IL-12, IL-15, and IFN-α trigger cytokine and perforin/granzymes secretion, respectively, while blockades of NKG2D and KCTD9 reduce inflammatory cytokine and cytotoxic component production. NCRs, Natural cytotoxic receptors (NCRs: NKp30, NKp44, NKp46); FasL, Fas ligand; TRAIL, Tumor necrosis factor related apoptosis inducing ligand; KCTD9, Potassium channel tetramerization domain.

**Figure 6:**
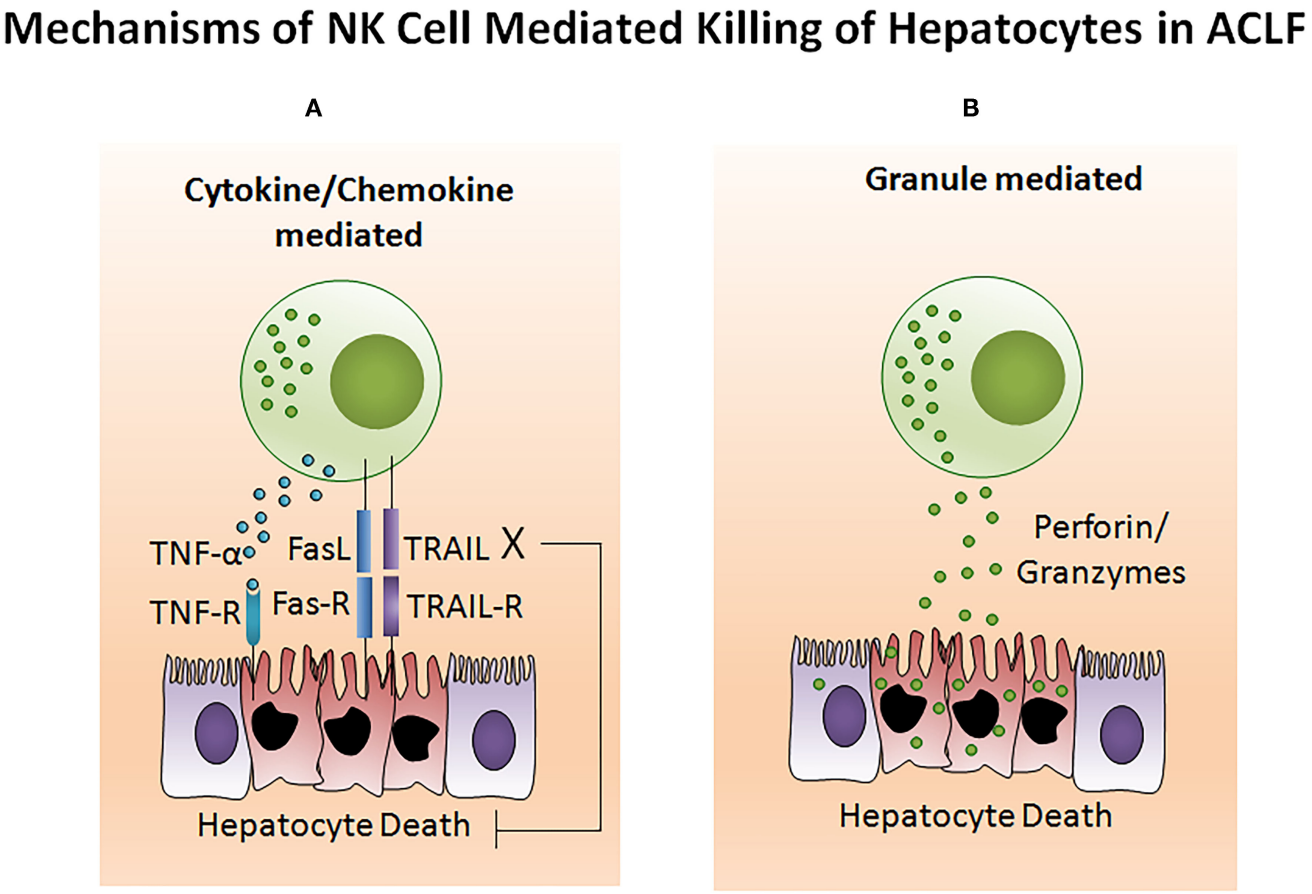
NK cell mediates hepatocyte death in ACLF. **(A)** Generally, NK cell-mediated cell death comprises extensive membrane blebbing, chromatin condensation, and nuclear DNA fragmentation resulting in apoptosis. TNF-α, TRAIL, and FasL belong to the TNF super family which are potential inducers for cell death. Activated NK cells produce a high amount of TNF-α, TRAIL, and FasL which sends an apoptotic signal to the hepatocytes expressing the corresponding receptors TNFR, TRAILR, and FasR. Transmission of the cytotoxic signal through the intracellular domain (death domain) of the TNF receptor leads to both apoptotic as well as necrotic cell death. However, FasL and TRAIL induces apoptotic cell death. FasL and TRAIL binds to its subsequent receptors. Ligand binding induces receptor oligomerization, followed by the recruitment of the adapter protein to the death domain and resulting in cell death. **(B)** Cytotoxic granules of NK cells contain several proteins including the pore-forming protein known as perforin and serine proteases termed as granzymes. Upon activation, NK cells release the cytotoxic granules through the process of exocytosis. After release, perforin create pores in the hepatocyte membrane which facilitates the release of granzymes into hepatocytes. These granzymes then induce hepatocyte death by proteolysis of the cytosolic and nuclear component through caspase-dependent as well as caspase-independent pathways. TNF-α, Tumor necrosis factor-α; TRAIL, TNF related apoptosis inducing ligand; FasL, Fas ligand.

The potency of the NK cell-mediated immune response is dependent on both its number and functional status. Several studies reported NK cell function in ACLF patients (105–108). Decreased NK cell numbers and cytotoxic CD56_dim_ CD16_bright_ NK cells have been observed in the circulation of HBV-ACLF patients (105). NK cell functions are regulated by activating cytotoxic (NKp30, NKp44, NKp46, and NKG2D) as well as inhibitory receptors CD158a (KIR2DL1) and CD158b (KIR2DL3). The expression of both activating as well as inhibitory receptors were found upregulated in ACLF patients (APASL ACLF) except for CD158b which was downregulated. Similarly, the augmented expression of peripheral natural cytotoxicity receptors (NCRs), NKp30 and NKp46, in HBV-ACLF patients showed a correlation with disease progression which was evaluated by the increased total bilirubin and prothrombin time activity (109). Moreover, NK cell mediated killing and cytotoxic activity and TNF-α production was severely decreased in HBV-ACLF (APASL ACLF) (108) which suggests that inhibitory receptors predominated over activating receptors without hampering NK cells degranulation and IFN-γ production. NK cell stimulation with IL-12 and IL-15 increased the production of TNF-α and IFN-γ, still the cytokine production was lowest in ACLF as compared to healthy control and chronic hepatitis B patients. The increased frequency of intrahepatic CD57+ CD3– NK cells contributed to hepatocyte necrosis in HBV-ACLF (APASL ACLF) through Fas/FasL and the NKG2D/NKG2D ligand pathway, leading to pathogenesis (108). NKG2D, a C lectin-like receptor of the NKG2 family, acts as an activating receptor and has the ability to trigger NK cell cytotoxicity, massively upregulating in the circulation and liver of HBV-ACLF patients (APASL ACLF) (107). In contrast to the previous data showing decreased IFN-γ and TNF-α+ NK cells, these data documented high peripheral and intrahepatic IFN-γ+ NK cells in HBV-ACLF patients. Also, IFN-γ, TNF-α, perforin, and granzymes were highest in serum. Further, NK cell stimulation with IFN-α not only upregulated NKG2D expression but also IFN-γ, TNF-α, perforin, and granzyme B production. Though the blockade of NKG2D leads to partial reduction of these cytokines and cytotoxic components, resulting in decreased NK cell cytotoxic function. This may suggest a role for NKG2D in NK cell-mediated immune inflammation (Figure 5). *In vitro* co-culture of NK cells with HepG2 cells transfected with wild-type HBVpCH-9/3093 plasmid DNA, which maintains stable HBV replication and HBs antigen expression, showed a slight decrease in HBV DNA and the HBs antigen level, however that was significantly inhibited after IFN-α treatment. Though the blockade of NKG2D moderately inhibited HBV replication and HBs antigens suggesting that during HBV-ACLF, NK cells act as a dual-edged sword. They regulate inflammation and liver injury by producing high amount of inflammatory cytokines and cytotoxic components besides limiting HBV replication and HBs antigen production. Peripheral NK cells also have higher tumor necrosis factor alpha related apoptosis inducing ligand (TRAIL) expression due to the abundance of circulating IL-6 and IL-8 in these patients (110). Increased TRAIL caused extensive apoptosis of hepatocytes which was further enhanced by IL-6 and IL-8 stimulation, signifying NK cell mediated toxicity through the TRAIL pathway, which was inhibited after the TRAIL blockade. NK cell activation induces hepatocyte death by both caspase-dependent as well as caspase-independent pathways (Figures 6A,B). KCTDN 9, a potassium channel tetramerization domain containing protein, acts as a potential inducer of NK cell mediated liver injury in HBV-ACLF (APASL ACLF) (111). Over expression of KCTD9 leads to a marked increase in the NK cell activation marker CD69, enhanced cytotoxicity, IFN-γ secretion, and a clear reduction in NKG2A receptor expression. The inhibition of KCTD9 decreased the NK cell cytotoxic function, suggesting KCTD9 as an essential component of the activation cascade.

## Innate Immune Targets For Therapeutics

As mentioned above, several innate immune cells and their functions play critical and complex roles in the progression of ACLF. Although innate immune pathways are essential for the clearance of infection, damaged cells, and cellular debris from tissue, their persistent activation leads to chronic liver inflammation, hepatic damage, and subsequently organ failure. Table 1 describes different innate immune targets which are crucial for therapeutic purposes. However, before targeting innate immune cells for the treatment of ACLF, it is equally important to identify a patient's current immune status. Identification of the exact immune phase of the patient will facilitate the application of immunotherapies and hence provide subsequent advancement in treatment of ACLF.

**Table 1:** Critical innate immune targets for therapeutics.

**Cell types**	**Key targets**	**Biological function/Outcome**	**Ref**
Neutrophils	CXCR1/CXCR2	Mediates pro-inflammatory cytokines and ROS production; induces apoptosis and necrosis in liver	(53)
	NGAL	Associated with systemic inflammation and liver failure	(72, 73)
Monocytes	MERTK	Induces systemic inflammatory response, associated with severity of intrahepatic, and extrahepatic diseases	(76)
	HLA-DR	Downregulation of monocyte function, predisposes recurrent infection leading to increased risk of mortality	(74, 112, 113)
Macrophages	sCD163	Associated with disease severity, independently correlates with short- and long-term mortality, induces hepatocyte differentiation, and liver regeneration	(86, 114, 115)
	CD163	Disease severity and prognosis	(87)
		Systemic inflammatory response	(115)
	MERTK	Severity of intrahepatic and extrahepatic diseases	(76)
NK cells	NKG2D	Inflammation	(107)
	TRAIL	Hepatocyte apoptosis	(110)
	KCTD9	NK cell activation and liver injury	(111)

## Cytokine Responses During ACLF

At present, one the major factors limiting the successful treatment of ACLF is the inadequate knowledge of the pathophysiology of the disease. Although, innate immune cells serve as a major contributor in the pathophysiology of ACLF; other inflammatory mediators including cytokines and chemokines play a critical role in mediating systemic inflammation and further worsen the condition of ACLF patients (EASL-CLIF ACLF) (116). Systemic circulatory dysfunction and exacerbation of systemic inflammation leads to organ failure(s). Increased levels of several cytokines including IFN-α2, IFN-γ, TNF-α, soluble TNF-αR1, soluble TNF-αR2, IL-2, IL-2R, IL-6, IL-8, IL-9, IL-10, IL-17, IL-23, IL-33, IL-35, TGF-β, soluble Fas antigen, macrophage chemotactic protein 1 (MCP-1), macrophage inflammatory protein(MIP)-1β, G-CSF, G-MCSF, vascular cell adhesion molecule-1, vascular endothelial growth factor, eotaxin, and HNA2 have been reported in ACLF (APASL and CANONIC ACLF) (53, 99, 117–123). The majority of these cytokines are related to the innate immune response such as leukocyte migration, particularly monocytes and macrophages, chemotaxis pathways, endothelial dysfunction, and subsequently induces systemic inflammation. Those cytokines which are involved in the innate immune response were significantly upregulated in ACLF patients than those without ACLF, suggesting that although the activation of both the innate and adaptive immune system occurs during ACLF, a dysregulated innate immune response might be a predominant mechanism in inducing systemic inflammation and disease progression in ACLF (123). Also, the assessment of the overall cytokine profile during ACLF reveals that not only proinflammatory molecules, but anti-inflammatory cytokines were also elevated, reflecting a generalized activation of cytokine cascade in these patients. However, the cytokine profile varies depending on the precipitating event including active alcoholism, acute alcoholic hepatitis, and bacterial infection. It is important to note that increased levels of G-CSF, G-MCSF, TNF-α, IFN-γ, and IL-6 participated in the process of emergency hematopoiesis, which develops in the setting of systemic inflammation (124, 125); therefore, the activation of these cytokines in ACLF (EASL-CLIF ACLF) possibly contributes to the leukocytosis and is associated with ACLF severity and patient prognosis (1, 126). Moreover, the dysregulation of IFN-γ not only induced systemic inflammation but impaired liver regeneration in ACLF patients, shown by the lower number of Ki67+ and PCNA+ hepatocytes in the presence of high IFN-γ (127). IFN-γ strongly attenuates liver regeneration and induces liver injury by activating STAT1 and p21 (128). The shift of the pro-regenerative pathway to the anti-regenerative pathway ultimately leads to liver failure in ACLF. Also, IFN-γ+ cells strongly are associated with TNF-α+ cells, which supports the concept that IFN-γ and TNF-α may act synergistically to induce liver injury during ACLF (129). The TNF-α signaling pathway induces apoptotic and necrotic cell death and is associated with other hepatocellular abnormalities and is most likely involved in the pathogenesis of ACLF.

Recently, the impact of IL-22 and the IL-22 binding protein (IL-22BP) have been studied in ACLF patients (EASL-CLIF ACLF) (130). A strong association between IL-22 serum levels and adverse outcomes of liver cirrhosis has been revealed. High levels of serum IL-22 and low ratios of IL-22BP/IL-22 are strongly associated with the presence of, or progression to, ACLF and mortality. Excessive secretion of IL-22BP can neutralize IL-22 *in vitro* and may prevent hepatoprotection likely in a context-specific manner, but may also prevent the adverse effects of IL-22. It has been shown that IL-22BP inhibits hepatic IL-22 signaling and the acute phase protein (130). Other data revealed the involvement of IL-22Fc therapy in liver regeneration by reprogramming the regenerative pathways and attenuation of bacterial infection that ameliorated ACLF, which suggests the therapeutic potential for IL-22 in these patients (127). IL-22Fc treatment upregulates the hepatic expression of multiple antibacterial genes in ACLF mice, contributing to the antibacterial effects of IL-22Fc. IL-22Fc is a recombinant fusion protein consisting of two human IL-22 molecules linked to an immunoglobulin constant region (IgG2-Fc) which is currently being examined in clinical trials for the treatment of severe alcoholic hepatitis. IL-22 is well-known for its hepatoprotective (131), pro-regenerative (132), and antimicrobial abilities (133), while ACLF is associated with hepatocyte damage, impaired liver regeneration, and bacterial infection; therefore, IL-22 treatment could benefit the problems that arise in ACLF. Despite ACLF being associated with elevated IL-22 and IL-22BP, several lines of evidence suggest that IL-22Fc therapy is still effective for patients with ACLF. Recently, a phase IIb clinical trial revealed IL-22Fc therapy as safe in patients with moderate and severe alcoholic hepatitis that improved the MELD score in these patients (134). Cytokines and chemokines have diverse functions that are not only restricted to hepatic injury and systemic inflammation. They also provide protection from liver injury, help in tissue healing and repair, and support liver regeneration. Modulation of the hepatic proinflammatory environment may provide novel strategies for the prevention and treatment of ACLF patients. In Table 2, we summarized cytokines and their functions which could be targeted for therapeutic interventions.

**Table 2:** Cytokines, critical target for immunotherapeutic in ACLF.

**Cytokines**	**Biological function/Outcome**	**Ref**
IFN-γ	Induces systemic inflammation, attenuates liver regeneration	(99, 127)
TNF-α	Induces systemic inflammation and liver injury, immunopathogenesis, encourages apoptotic, and necrotic cell death	(76, 129)
IL-1	Initiates inflammatory process and development of ACLF	(135)
IL-6	Induces systemic inflammatory pathways, is associated with disease severity, short term mortality	(34, 123)
IL-8	Neutrophil infiltration, liver inflammation, short term mortality	(51, 53, 123)
IL-10	Associated with poor prognosis and mortality	(136)
IL-17	Associated with clinical disease severity markers including INR and MELD, mortality, recruits proinflammatory cells, increases the expression of profibrogenic factor TGF-β, and inflammatory cytokines IL-6 and IL-23	(121, 136, 137)
IL-22	Hepatoprotective, supports liver regeneration, attenuates bacterial infection	(127, 130)
IL-23	Associated with clinical disease severity markers MELD, INR, prothrombin time, total bilirubin, and mortality	(101, 121)
G-CSF and G-MSCF	Reduces IFN-γ production, mobilizes CD34+ cells, improves clinical disease severity indices and survival in ACLF patients, reduces short term mortality, activates progenitors to promote differentiation and activation of monocytes and neutrophils	(36, 46, 99, 138–141)
TGF-β	Liver injury, disease severity, and poor survival in ACLF	(122)
VCAM-1, ICAM-1	Inflammation, short term mortality	(36)

## Conclusion

The present knowledge and understanding of the innate immune response of ACLF patients is limited. There are a number of specific issues left somewhat ambiguous, like whether a dysregulated innate immune response is the cause or outcome of ACLF. Few studies outlined abnormal innate immunity of ACLF patients which is associated with systemic inflammation, disease progression, and pathogenesis; however extensive research is required to find out the mechanisms responsible for disease severity and high mortality in these severely ill patients. In this review we elaborated several defects in innate immune cells and abnormal cytokine responses that compromise host immunity, make them susceptible to secondary infections, and cause systemic inflammation and further complications increasing the risk of high mortality. Techniques for identifying an accurate immune phase of ACLF patients whether it is the inflammatory phase or the immunosuppressive phase and distinguishing precise defects in innate immune cells will promote the development and application of dynamic new immunotherapies, which will help in the management of these diseases.

## Author Contributions

AK conceptualized, wrote the manuscript, and designed the illustrations. SK edited the manuscripts. All authors contributed to the article and approved the submitted version.

## Conflict of Interest

The authors declare that the research was conducted in the absence of any commercial or financial relationships that could be construed as a potential conflict of interest.

## Abbreviations

ACLF: Acute-on-chronic liver failure
ALF: Acute liver failure
CLD: Chronic liver disease
SIRS: Systemic inflammatory response syndrome
CARS: Compensatory inflammatory response syndrome
INR: International normalized ratio
CTP: Child-Turcotte-Pugh
MELD: Model for end stage liver disease
SOFA: Sequential organ failure assessment
CXCR: Chemokine receptor
CXCL: Chemokine ligand
NLR: Neutrophil to lymphocyte ratio
KCs: Kupffer cells
NGAL: Neutrophil gelatinase-associated lipocalin
MERTK: MER receptor tyrosine kinase
TRAIL: TNF-α related apoptosis inducing ligand
KCTDN9: Potassium channel tetramerization domain
IL: Interleukin.
